# Association of low‐grade albuminuria and triple‐line pattern as markers of early peripheral vascular remodeling in adolescents with type 1 diabetes

**DOI:** 10.1111/jdi.70283

**Published:** 2026-03-09

**Authors:** Jung‐Chi Hsu, Fu‐Sung Lo, Jiann‐Shing Jeng, Wen‐Yu Tsai, Sandy Huey‐Jen Hsu, Hsiang Yang, Tien‐Jyun Chang, Ta‐Chen Su

**Affiliations:** ^1^ Department of Internal Medicine National Taiwan University Hospital, Jinshan Branch New Taipei City Taiwan; ^2^ Division of Cardiology, Department of Internal Medicine National Taiwan University Hospital and College of Medicine Taipei Taiwan; ^3^ Division of Pediatric Endocrinology, Department of Pediatrics Chang Gung Memorial Hospital, Chung Gung University College of Medicine Taoyuan City Taiwan; ^4^ Department of Neurology National Taiwan University Hospital Taipei Taiwan; ^5^ Department of Pediatrics National Taiwan University College of Medicine Taipei Taiwan; ^6^ Department of Laboratory Medicine National Taiwan University Hospital, National Taiwan University College of Medicine Taipei Taiwan; ^7^ Department of Dentistry National Taiwan University Hospital Taipei Taiwan; ^8^ Division of Endocrinology and Metabolism, Department of Internal Medicine National Taiwan University Hospital Taipei Taiwan; ^9^ School of Medicine National Taiwan University College of Medicine Taipei Taiwan; ^10^ Division of Endocrinology and Metabolism, Department of Internal Medicine National Taiwan University Hospital Yun‐Lin Branch Douliu Yun‐Lin Taiwan; ^11^ Division of Cardiology, Department of Internal Medicine Tungs' Taichung MetroHarbor Hospital Taichung Taiwan; ^12^ Institute of Environmental and Occupational Health Sciences National Taiwan University College of Public Health Taipei Taiwan; ^13^ School of Post‐Baccalaureate Medicine National Chung Hsing University College of Medicine Taichung Taiwan

**Keywords:** Microalbuminuria, Triple‐line pattern, Type 1 diabetes

## Abstract

**Background:**

Adolescents with type 1 diabetes (T1D) face premature atherosclerosis, but standard carotid intima‐media thickness (IMT) may lack sensitivity for the earliest vascular injuries. Since urinary albumin‐to‐creatinine ratio (UACR) reflects systemic endothelial dysfunction, we investigated the triple‐line pattern (TLP) as a marker of peripheral remodeling and its dose–response relationship with low‐grade albuminuria.

**Methods:**

The Subclinical Cardiovascular Disease in Type 1 Diabetes Mellitus (SCVD T1DM) cohort study prospectively recruited 283 adolescents with T1D and 106 age‐ and sex‐matched controls. IMT was measured at the common carotid artery and common femoral artery (CFA) using high‐resolution ultrasound. Logistic regression tested categorical UACR thresholds, and restricted cubic spline models evaluated the continuous dose–response association between UACR and TLP.

**Results:**

TLP prevalence was higher in type 1 diabetes than in controls (63.9% vs 49.1%, *P* = 0.008). While type 1 diabetes was associated with carotid thickening, TLP was linked to structural changes in the lower extremities. TLP‐positive participants had greater mean CFA IMT (0.56 ± 0.06 vs 0.44 ± 0.06 mm, *P* < 0.001). UACR ≥15 mg/g independently predicted TLP (odds ratio 2.83, 95% confidence interval 1.01–7.92, *P* = 0.048), whereas UACR ≥30 mg/g was not significant. Spline analysis showed no significant nonlinearity (*P* for nonlinearity = 0.424), with risk increasing above approximately 15 mg/g.

**Conclusions:**

TLP is a prevalent marker of incipient peripheral vascular remodeling in adolescents with type 1 diabetes without macroalbuminuria. A UACR ≥15 mg/g identifies subclinical vascular risk below conventional thresholds, supporting TLP as a potential tool for refined early risk stratification in this population.

## Graphical abstract

In adolescents with type 1 diabetes, the arterial triple‐line pattern (TLP) signals early lower extremity vascular remodeling. Low‐grade albuminuria (UACR ≥15 mg/g) independently predicts TLP, supporting the combined use of these markers to identify subclinical atherosclerosis.

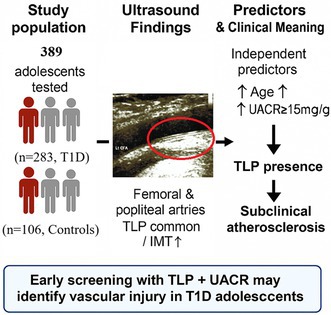

## INTRODUCTION

Type 1 diabetes diagnosed during childhood is associated with a significantly elevated risk of premature atherosclerotic cardiovascular disease (ASCVD)^1^, ^2^, ^3^. While glycemic management has improved, intensive glucose control alone is often insufficient to prevent macrovascular complications. The increasing incidence of type 1 diabetes in the youth population necessitates the identification of early vascular risk markers to enable intervention before the development of irreversible injury^4^, ^5^.

Early subclinical atherosclerosis is traditionally evaluated using carotid intima‐media thickness (IMT), which is a quantitative marker associated with future cardiovascular morbidity^6^, ^7^. Evidence indicates that lower extremity arteries, especially the femoral artery, exhibit heightened vulnerability to diabetes‐related metabolic stress and may demonstrate structural alterations prior to the carotid territory^8^, ^9^. Beyond traditional geometric thickening measured by IMT, the triple‐line pattern (TLP) has emerged as a novel qualitative ultrasonographic marker. TLP is characterized by the distinct visualization of the internal elastic lamina within the intima‐media complex. This structural feature is hypothesized to represent an incipient phase of vascular remodeling that occurs before a measurable quantitative increase in IMT is detectable^10^, ^11^, ^12^.

Although TLP is linked to cardiovascular risk factors in the general population, its clinical significance and vascular distribution in adolescents with type 1 diabetes remain to be elucidated. Furthermore, while urinary albumin excretion is a robust indicator of generalized endothelial damage, the association between TLP and incipient renal injury remains poorly understood. This is particularly relevant in the context of low‐grade urinary albumin excretion among patients who have not yet progressed to macroalbuminuria. To date, no systematic evaluation of TLP has been conducted in adolescents with type 1 diabetes, a population predisposed to accelerated vascular aging.

The present study aimed to explore the prevalence of early atherosclerosis, especially the presence of TLP, in adolescents with type 1 diabetes, and to evaluate its association with IMT across various vascular territories as well as early urinary albumin excretion.

## METHODS

### Study population

The Subclinical Cardiovascular Disease in Type 1 Diabetes Mellitus (SCVD T1DM) cohort study was conducted in Taiwan. We prospectively recruited patients with type 1 diabetes and nondiabetic siblings who were matched for age and sex. The diagnosis of type 1 diabetes was established based on the criteria of the American Diabetes Association^13^. To focus the investigation on the subclinical stages of vascular injury, participants with macroalbuminuria, defined as a urine albumin‐to‐creatinine ratio (UACR) exceeding 300 mg/g, were excluded from the analysis. Recruitment was facilitated by the Taiwan Association for Diabetic Children (TADC) as well as pediatric endocrinologists at National Taiwan University Hospital and Chang Gung Memorial Hospital. The study protocol received approval from the Institutional Review Board of National Taiwan University Hospital (IRB No: 201205043RIB). Written informed consent was obtained from all participants or their legal guardians.

All participants underwent standardized clinical assessments including anthropometric measurements, blood pressure monitoring, and fasting laboratory evaluations for glucose, HbA1c, lipids, creatinine, hs‐CRP, and liver enzymes. First morning urine samples were collected for routine chemistry and determination of the UACR.

### Vascular measurements

Carotid IMT was evaluated at the common carotid artery (CCA), carotid bulb, and internal carotid artery using a high‐resolution B‐mode ultrasound system (GE Vivid i, Horten, Norway). IMT was defined as the distance between the lumen‐intima and media‐adventitia interfaces on the far wall. Automated edge detection software was utilized to calculate mean and maximal IMT from at least 150 measurements per segment to ensure high reproducibility.

Lower extremity IMT was assessed at the common femoral artery (CFA), popliteal, anterior tibial, and posterior tibial arteries. The TLP was defined as the sonographic visualization of a third echogenic line within the intima‐media complex, which corresponds to the internal elastic lamina^10^, ^11^, ^12^. A participant was considered TLP‐positive if the pattern was identified in at least one segment of the femoral or popliteal arteries.

All images were stored in DICOM format and reviewed offline by two independent observers who were blinded to the clinical status of the participants. In addition to IMT and TLP, arterial wall morphology was assessed for focal plaque formation, and Doppler ultrasound was employed to evaluate arterial flow profiles.

### Statistical analysis

Continuous variables were expressed as mean and standard deviation, while categorical variables were presented as frequencies and percentages. Comparisons between groups were performed using the Student *t*‐test for continuous variables and the Chi‐square test for categorical variables. Multivariable logistic regression analysis was conducted using two distinct strategies to evaluate the determinants of the TLP. To identify independent predictors of TLP, variables that demonstrated significant associations in the univariable analyses were subsequently entered into a multivariable model. The results were reported as odds ratios (OR) and 95% confidence intervals (CI).

Furthermore, to evaluate the robust association between specific urinary UACR thresholds and the presence of TLP, three sequential adjustment models were utilized. Model 1 was the crude, unadjusted model. Model 2 was adjusted for demographic factors, including age and gender. Model 3, the fully adjusted model, further included body mass index (BMI), HbA1c, total cholesterol (TCHO), high‐density lipoprotein (HDL), low‐density lipoprotein (LDL), triglycerides (TG), small dense low‐density lipoprotein (sd‐LDL), advanced glycation end products (AGEs), and high‐sensitivity C‐reactive protein (hs‐CRP). These potential confounders were selected based on their established clinical relevance to atherosclerotic risk in patients with type 1 diabetes. To evaluate the linear relationship between urinary albumin excretion and arterial wall thickness, UACR values underwent log‐transformation to normalize the distribution. Linear regression models were then utilized to assess the correlation between log‐transformed UACR and the mean IMT of the carotid and femoral arteries.

The dose–response association between UACR and the risk of TLP was assessed using restricted cubic spline models. These models were implemented with three knots positioned at the 10th, 50th, and 90th percentiles of the UACR distribution. A nonlinear association was evaluated using the Wald test. The reference value for calculating log‐odds was set at the median UACR of the study population.

All statistical analyses were performed using SPSS software version 26.0 (IBM Corp, Armonk, NY) and R software version 4.3 (R Development Core Team, Vienna, Austria). A two‐sided *P*‐value of less than 0.05 was considered to indicate statistical significance.

## RESULTS

### Baseline characteristics of the study population

A total of 389 participants were included in the final analysis, comprising 283 individuals with type 1 diabetes and 106 age‐ and sex‐matched nondiabetic siblings. To focus on the subclinical stages of vascular injury, three participants with type 1 diabetes were excluded due to macroalbuminuria (UACR values of 1252.52, 990.98, and 540.07 mg/g). Among the remaining 384 participants with available renal data, the overall mean UACR was 8.99 mg/g (median 4.26 mg/g; interquartile range 2.49–8.26 mg/g), ranging from 0.01 to 193.24 mg/g. Within the T1D cohort, the mean age at diagnosis was 8.59 ± 4.07 years. The average disease duration was 7.26 ± 4.84 years, with a median of 6.69 years and an interquartile range of 3.41–10.18 years (range 0.1–26.95 years). Detailed clinical and demographic characteristics of the type 1 diabetes and control groups are presented in Table 1.

**Table 1:** Baseline clinical characteristics and early vascular markers in adolescents with type 1 diabetes and nondiabetic controls

Variable	T1D (*n* = 283)	Sibling controls (*n* = 106)	*P*‐value
Age, years	15.76 ± 4.81	16.76 ± 5.99	0.089
Duration of diabetes diagnosis, years	7.26 ± 4.8	–	–
Male, *n* (%)	118 (41.7)	38 (35.8)	0.295
BMI, kg/m^2^	20.17 ± 3.29	20.95 ± 4.29	0.057
Fasting plasma glucose (mg/dL)	184.81 ± 78.81	98.43 ± 7.61	<0.001
HbA1c (%)	8.35 ± 1.86	5.33 ± 0.30	<0.001
AGEs	1.82 ± 1.04	1.86 ± 0.87	0.732
sd‐LDL, mg/dL	20.27 ± 14.25	19.21 ± 9.40	0.478
TCHO, mg/dL	176.29 ± 35.04	173.82 ± 34.14	0.534
TG, mg/dL	68.88 ± 54.41	76.21 ± 39.81	0.206
HDL, mg/dL	67.30 ± 13.67	58.94 ± 11.33	<0.001
LDL, mg/dL	94.86 ± 31.35	101.28 ± 31.41	0.073
UA	4.47 ± 1.11	5.50 ± 1.58	<0.001
ALT	14.51 ± 19.44	15.46 ± 11.74	0.635
hs‐CRP, mg/L	0.59 ± 1.22	0.11 ± 0.22	<0.001
SBP, mmHg	109.69 ± 12.71	108.28 ± 14.58	0.350
DBP, mmHg	58.88 ± 7.26	58.67 ± 8.20	0.804
PP, mmHg	50.82 ± 9.20	49.62 ± 9.84	0.263
Renal function			
BUN	13.03 ± 3.02	12.49 ± 2.95	0.118
Creatinine	0.82 ± 0.15	0.85 ± 0.17	0.114
eGFR	84.55 ± 11.50	80.98 ± 10.88	0.006
UACR, mg/g	9.76 ± 20.89	6.96 ± 12.35	0.195
UACR ≥30 mg/g	13 (4.6)	4 (3.8%)	0.701
UACR ≥15 mg/g	32 (11.3%)	6 (5.7%)	0.086
Common carotid artery (CCA), mm			
Left CCA	0.42 ± 0.04	0.43 ± 0.05	0.552
Right CCA	0.43 ± 0.04	0.43 ± 0.04	0.686
Mean CCA	0.42 ± 0.03	0.42 ± 0.04	0.203
Max CCA	0.53 ± 0.04	0.52 ± 0.05	**0.001**
Carotid bulb, mm			
Left bulb	0.44 ± 0.06	0.42 ± 0.06	**0.023**
Right bulb	0.44 ± 0.06	0.43 ± 0.06	0.091
Mean bulb	0.44 ± 0.05	0.43 ± 0.05	0.016
Max bulb	0.52 ± 0.06	0.50 ± 0.06	0.011
Internal carotid artery (ICA), mm			
Left ICA	0.37 ± 0.05	0.37 ± 0.05	0.503
Right ICA	0.38 ± 0.05	0.39 ± 0.05	0.510
Mean ICA	0.38 ± 0.04	0.38 ± 0.04	0.991
Max ICA	0.45 ± 0.05	0.44 ± 0.05	0.019
Common femoral artery (CFA), mm			
Left CFA	0.55 ± 0.16	0.52 ± 0.17	0.100
Right CFA	0.58 ± 0.16	0.56 ± 0.17	0.346
Mean CFA	0.57 ± 0.14	0.54 ± 0.15	0.135
Max CFA	0.66 ± 0.15	0.63 ± 0.15	0.128
Popliteal artery, mm			
Left pop A	0.44 ± 0.06	0.44 ± 0.07	0.888
Right pop A	0.43 ± 0.07	0.43 ± 0.06	0.921
Mean pop A	0.43 ± 0.05	0.43 ± 0.05	0.962
Max pop A	0.51 ± 0.06	0.50 ± 0.06	0.051
TLP	181 (63.9)	52 (49.1|)	0.008

Abbreviations: AGEs, advanced glycation end products; ALT, alanine transaminase; BMI, body mass index; BUN, blood urea nitrogen; CCA, common carotid artery; CFA, common femoral artery; DBP, diastolic blood pressure; FPG, fasting plasma glucose; HbA1c, glycated hemoglobin; HDL, high‐density lipoprotein; hs‐CRP, high‐sensitivity C‐reactive protein; ICA, internal carotid artery; LDL, low‐density lipoprotein; Pop A, popliteal artery; PP, pulse pressure; SBP, systolic blood pressure; sd‐LDL, small dense low‐density lipoprotein; T1D, type 1 diabetes; TCHO, total cholesterol; TG, triglycerides; TLP, triple‐line pattern; UA, uric acid; UACR, urinary albumin‐to‐creatinine ratio.

The prevalence of the TLP was significantly higher in the type 1 diabetes group compared with the control group, representing 63.9% vs 49.1%, *P* = 0.008. Patients with type 1 diabetes exhibited significantly higher fasting plasma glucose (184.81 ± 78.81 vs 98.43 ± 7.61 mg/dL, *P* < 0.001), HbA1c (8.35 ± 1.86 vs 5.33 ± 0.30%, *P* < 0.001), and hs‐CRP (0.59 ± 1.22 vs 0.11 ± 0.22 mg/L, *P* < 0.001). Regarding renal function, the type 1 diabetes group showed a significantly higher eGFR (84.55 ± 11.50 vs 80.98 ± 10.88 mL/min/1.73 m^2^, *P* = 0.006) and lower uric acid levels (4.47 ± 1.11 vs 5.50 ± 1.58 mg/dL, *P* < 0.001) compared with controls.

### Vascular parameters and IMT in relation to T1D and TLP

**Figure 1:**
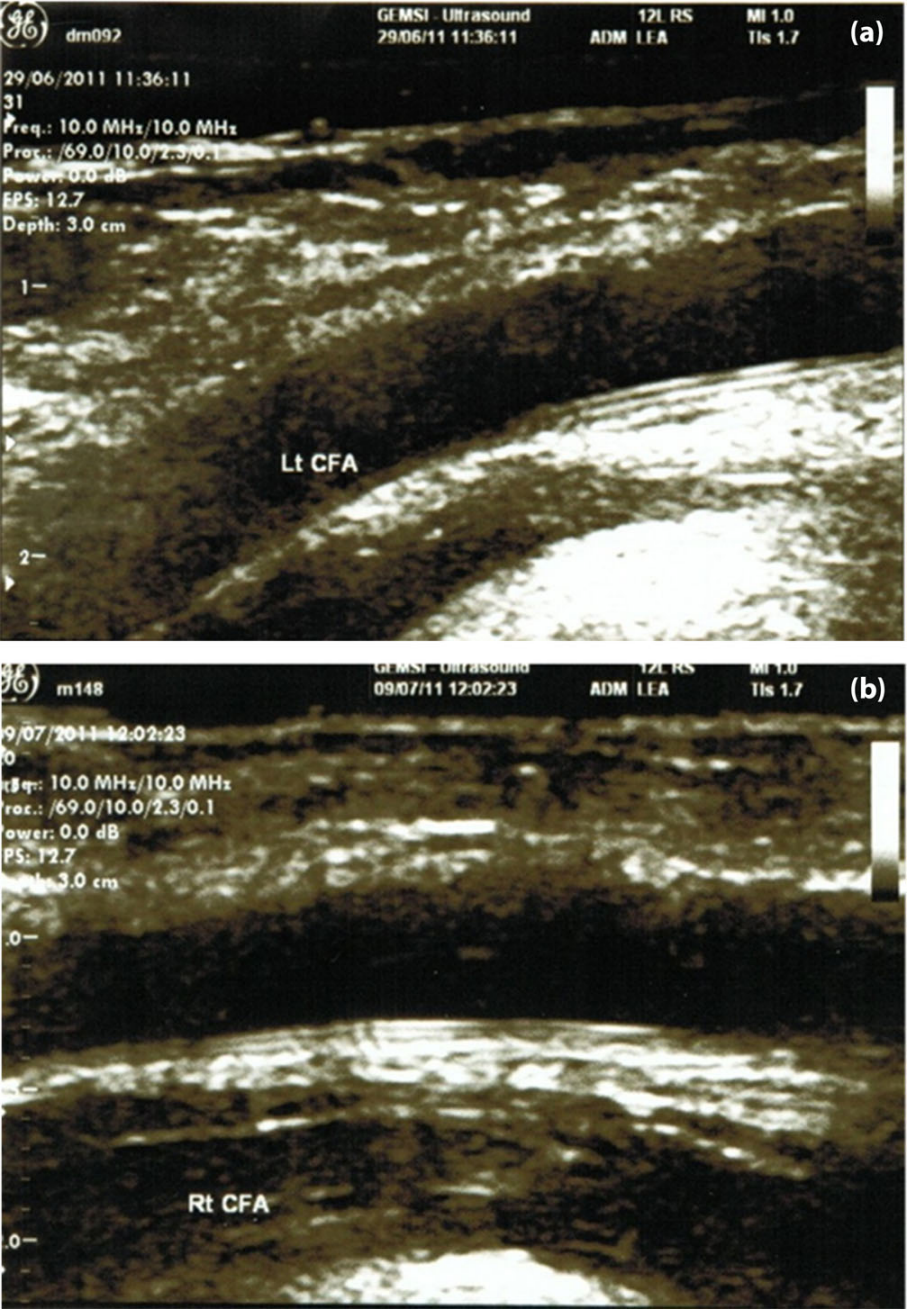
Representative ultrasound images of the triple‐line pattern (TLP) in lower extremity arteries of adolescents with type 1 diabetes. (a) A 21‐year‐old woman with T1D for 13.3 years (BMI 17.0 kg/m^2^, plasma glucose 166 mg/dL, HbA1c 6.5%) demonstrated TLP in the left CFA, but no TLP in the carotid artery. (b) A 18‐year‐old man with type 1 diabetes for 4.5 years (BMI 19.6 kg/m^2^, plasma glucose 77 mg/dL, HbA1c 9.7%) exhibited TLP in both the right common femoral and popliteal arteries, whereas the carotid artery was free of TLP.

Figure 1 provides representative cases of TLP in the lower extremities of adolescents with type 1 diabetes. Compared with controls, adolescents with type 1 diabetes showed modest but statistically significant increases in several carotid IMT parameters, including maximum CCA IMT (0.53 ± 0.04 vs 0.52 ± 0.05 mm, *P* = 0.001), left bulb IMT (0.44 ± 0.06 vs 0.42 ± 0.06 mm, *P* = 0.023), mean bulb IMT (0.44 ± 0.05 vs 0.43 ± 0.05 mm, *P* = 0.016), maximum bulb IMT (0.52 ± 0.06 vs 0.50 ± 0.06 mm, *P* = 0.011), and maximum ICA IMT (0.45 ± 0.05 vs 0.44 ± 0.05 mm, *P* = 0.019). In contrast, lower extremity IMT measurements were comparable between groups. The mean CFA IMT was 0.57 ± 0.14 mm in the type 1 diabetes group and 0.54 ± 0.15 mm in controls (*P* = 0.135), while mean popliteal artery IMT was 0.43 ± 0.05 mm in both groups (*P* = 0.962). These findings indicate that adolescents with type 1 diabetes already exhibit early carotid thickening, whereas generalized femoral or popliteal IMT increases are not yet evident at this stage.

A subanalysis stratified by TLP status is given in Table 2. In contrast to the between‐group carotid differences, TLP was primarily associated with structural changes in the lower extremity arteries. Carotid IMT did not differ significantly between participants with and without TLP across measured segments, including mean CCA IMT (0.42 ± 0.03 vs 0.43 ± 0.03 mm, *P* = 0.064). By contrast, participants with TLP had substantially greater CFA IMT, including higher mean CFA IMT (0.56 ± 0.06 vs 0.44 ± 0.06 mm, *P* < 0.001) and higher maximum CFA IMT (0.64 ± 0.07 vs 0.52 ± 0.07 mm, *P* < 0.001). Collectively, these results suggest that TLP may capture an early pattern of lower extremity vascular remodeling that is not fully reflected by carotid IMT in this adolescent cohort.

**Table 2:** Vascular parameters and intima‐media thickness in participants with type 1 adolescents

Vascular parameters (mm)	With TLP (+), *n* = 87	Without TLP, *n* = 92	*P*‐value
Common carotid artery (CCA)			
Left CCA	0.42 ± 0.04	0.42 ± 0.05	0.656
Right CCA	0.43 ± 0.04	0.44 ± 0.04	0.150
Mean CCA	0.42 ± 0.03	0.43 ± 0.03	0.064
Max CCA	0.53 ± 0.04	0.53 ± 0.04	0.519
Carotid bulb			
Left bulb	0.42 ± 0.06	0.44 ± 0.07	0.239
Right bulb	0.44 ± 0.06	0.43 ± 0.06	0.147
Mean bulb	0.43 ± 0.05	0.43 ± 0.05	0.926
Max bulb	0.51 ± 0.06	0.51 ± 0.06	0.685
Internal carotid artery (ICA)			
Left ICA	0.37 ± 0.05	0.37 ± 0.05	0.566
Right ICA	0.39 ± 0.06	0.38 ± 0.05	0.197
Mean ICA	0.38 ± 0.04	0.37 ± 0.04	0.26
Max ICA	0.45 ± 0.05	0.45 ± 0.05	0.614
Common femoral artery (CFA)			
Left CFA	0.53 ± 0.15	0.43 ± 0.06	<0.001
Right CFA	0.58 ± 0.13	0.45 ± 0.07	<0.001
Mean CFA	0.56 ± 0.06	0.44 ± 0.06	<0.001
Max CFA	0.64 ± 0.07	0.52 ± 0.07	<0.001
Popliteal artery			
Left popliteal	0.44 ± 0.05	0.43 ± 0.05	0.178
Right popliteal	0.41 ± 0.05	0.42 ± 0.05	0.200
Mean popliteal	0.42 ± 0.04	0.42 ± 0.04	0.938
Max popliteal	0.50 ± 0.05	0.49 ± 0.05	0.096

Abbreviations: CCA, common carotid artery; CFA, common femoral artery; ICA, internal carotid artery; TLP, triple‐line pattern.

### Independent predictors of TLP and low‐grade albuminuria

The results of the logistic regression analyses for predictors of TLP are summarized in Table 3. In the univariable models, type 1 diabetes status (OR 1.84, 95% CI: 1.17–2.90, *P* = 0.008), age (OR 1.08, 95% CI: 1.04–1.13, *P* < 0.001), HbA1c (OR 1.15, 95% CI: 1.04–1.28, *P* = 0.007), eGFR (OR 1.02, 95% CI: 1.00–1.04, *P* = 0.031), and UACR (OR 1.02, 95% CI: 1.00–1.05, *P* = 0.037) were all significantly associated with the presence of TLP. In the multivariable model, age remained a highly significant independent predictor (OR 1.09, 95% CI: 1.04–1.14, *P* < 0.001), while type 1 diabetes status showed a trend toward significance (OR 1.78, 95% CI: 0.94–3.39, *P* = 0.078).

**Table 3:** Multivariable logistic regression analysis for independent predictors of triple‐line pattern

Type	Univariable logistic regression		Multivariable logistic regression	
	Odds ratio (95% CI)	*P*‐value	Odds ratio (95% CI)	*P*‐value
T1DM, %	1.84 (1.17–2.90)	0.008	1.78 (0.94–3.39)	0.078
Age, years	1.08 (1.04–1.13)	<0.001	1.09 (1.04–1.14)	<0.001
Duration of diabetes diagnosis, years	1.04 (0.98–1.10)	0.168		
Male, %	0.75 (0.50–1.14)	0.173	–	–
BMI, kg/m^2^	1.01 (0.96–1.07)	0.628	–	–
FPG, mg/dL	1.00 (0.99–1.00)	0.482	–	–
HbA1c, %	1.15 (1.04–1.28)	0.007	1.03 (0.89–1.19)	0.706
AGEs	1.02 (0.83–1.26)	0.831	–	–
sd‐LDL, mg/dL	1.00 (0.99–1.02)	0.633	–	–
TCHO, mg/dL	1.00 (0.98–1.01)	0.670	–	–
TG, mg/dL	1.00 (1.00–1.01)	0.840	–	–
HDL, mg/dL	1.00 (0.99–1.02)	0.927	–	–
LDL, mg/dL	1.00 (0.99–1.00)	0.417	–	–
eGFR	1.02 (1.00–1.04)	0.031	1.01 (0.98–1.03)	0.667
hs‐CRP, mg/L	1.28 (0.98–1.67)	0.072	–	–
UACR, mg/g	1.02 (1.00–1.05)	0.037	1.02 (0.99–1.04)	0.062
SBP, mmHg	1.01 (0.99–1.03)	0.249	–	–
DBP, mmHg	1.02 (0.99–1.05)	0.115	–	–
PP, mmHg	1.00 (0.98–1.03)	0.719	–	–

Abbreviations: AGEs, advanced glycation end products; ALT, alanine transaminase; BMI, body mass index; BUN, blood urea nitrogen; FPG, fasting plasma glucose; HbA1c, glycated hemoglobin; HDL, high‐density lipoprotein; hs‐CRP, high‐sensitivity C‐reactive protein; LDL, low‐density lipoprotein; sd‐LDL, small dense low‐density lipoprotein; T1D, type 1 diabetes; TCHO, total cholesterol; TG, triglycerides; UA, uric acid; UACR, urinary albumin‐to‐creatinine ratio.

To further examine the influence of albuminuria thresholds, we tested different UACR cutoffs using a hierarchical adjustment strategy. The results are summarized in Table 4. Among control siblings, UACR thresholds of 15 mg/g or higher and 30 mg/g or higher were not associated with the presence of TLP in any model. In contrast, in the type 1 diabetes group, a UACR of 15 mg/g or higher demonstrated a robust and consistent association with TLP. In the unadjusted model (Model 1), the odds ratio was 2.73 (95% CI: 1.08–6.87, *P* = 0.033). The association remained significant after adjustment for age and sex (Model 2; OR 2.59, 95% CI: 1.01–6.67, *P* = 0.048) and persisted in the fully adjusted model incorporating metabolic and inflammatory markers (Model 3; OR 2.83, 95% CI: 1.01–7.92, *P* = 0.048). By contrast, the conventional cutoff of UACR 30 mg/g or higher was not statistically significant in the fully adjusted model (OR 2.44, 95% CI: 0.44–13.47, *P* = 0.307).

**Table 4:** Association between urinary albumin‐to‐creatinine ratio (UACR) thresholds and triple‐line pattern

UACR, mg/g	Model 1		Model 2		Model 3	
	Odds ratio (95% CI)	*P*‐value	Odds ratio (95% CI)	*P*‐value	Odds ratio (95% CI)	*P*‐value
Control siblings						
*Category 1*						
UACR <15	1		1			
UACR ≥15	2.17 (0.38–12.37)	0.384	2.21 (0.37–13.18)	0.386	2.18 (0.32–14.92)	0.427
*Category 2*						
UACR <30	1		1		1	
UACR ≥30	3.25 (0.33–32.24)	0.315	2.84 (0.60–13.39)	0.188	2.96 (0.26–32.99)	0.379
T1D						
*Category 1*						
UACR <15	1		1		1	
UACR ≥15	2.73 (1.08–6.87)	0.033	2.59 (1.01–6.67)	0.048	2.83 (1.01–7.92)	0.048
*Category 2*						
UACR <30	1		1		1	
UACR ≥30	3.28 (0.71–15.10)	0.127	2.84 (0.60–13.39)	0.188	2.44 (0.44–13.47)	0.307

Model 1: Crude; Model 2: Adjust age and gender; Model 3: Adjust age, gender, BMI, HbA1c, TCHO, HDL, LDL, TG, sd‐LDL, AGEs, hs‐CRP. UACR, Urine albumin‐to‐creatinine ratio.

**Figure 2:**
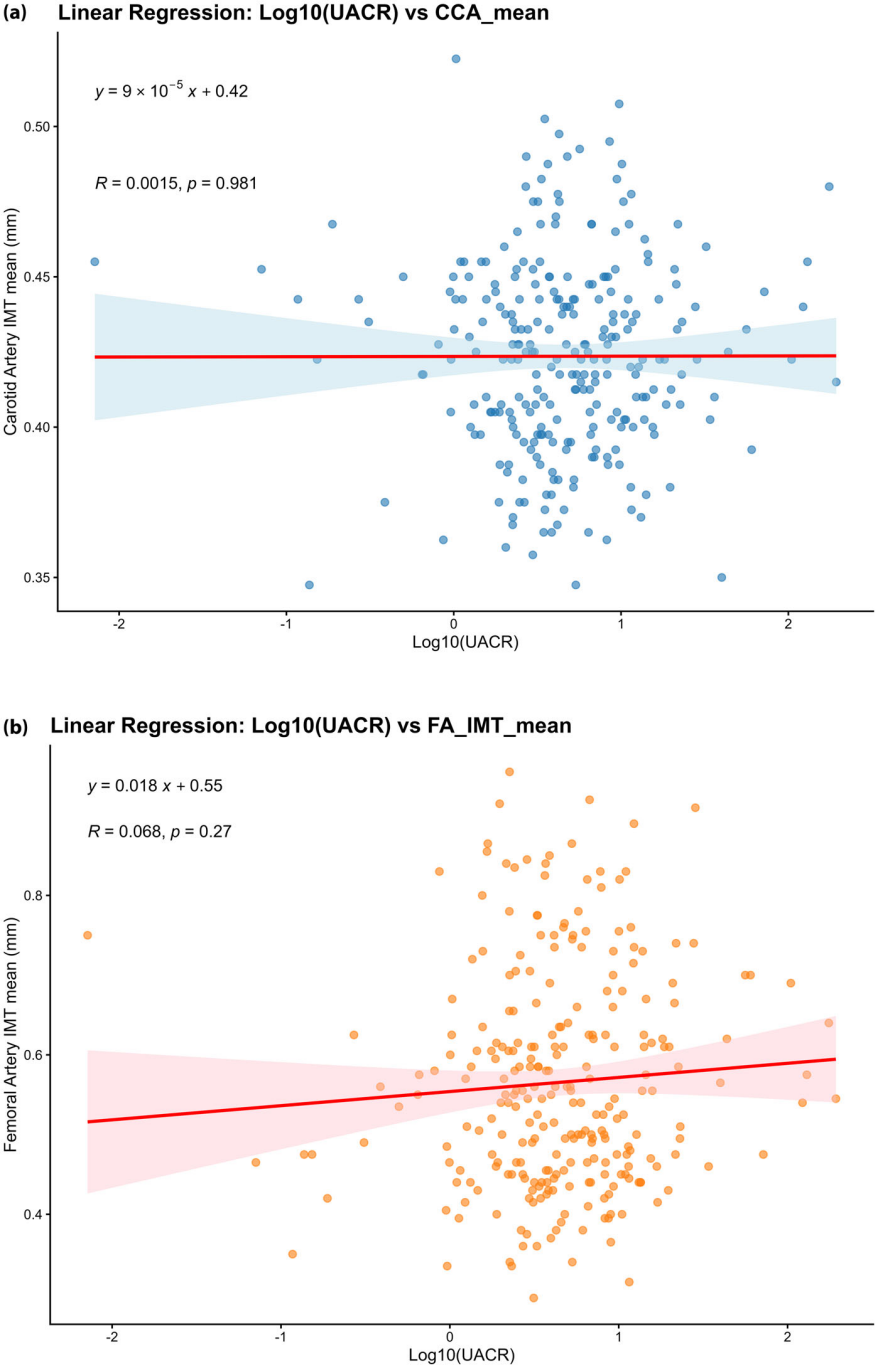
Linear correlation between urinary albumin excretion and arterial intima‐media thickness in type 1 diabetes. Scatter plots demonstrating the relationship between log‐transformed urinary albumin‐to‐creatinine ratio and the mean intima‐media thickness of the (a) common carotid artery and (b) common femoral artery.

**Figure 3:**
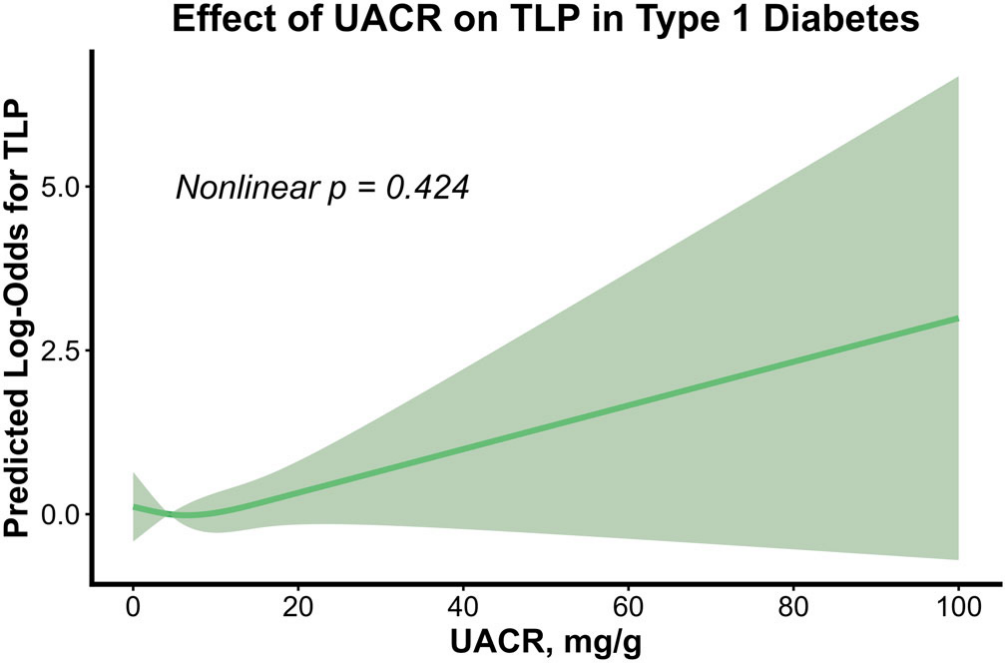
A dose–response relationship between urine albumin‐to‐creatinine ratio (UACR) and the risk of triple‐line pattern (TLP) in type 1 diabetes. Restricted cubic spline analysis illustrating a linear association with no significant nonlinearity between continuous UACR levels and the predicted log‐odds of TLP in the T1D cohort. A sharp upward trend emerged when UACR exceeded 15 mg/g, indicating an elevated risk of early arterial changes even below the conventional threshold for microalbuminuria (≥30 mg/g).

Figure 2 illustrates the linear correlation between log‐transformed UACR and arterial IMT. The restricted cubic spline analysis in Figure 3 demonstrated a linear association between continuous UACR levels and the risk of TLP (*P* for nonlinearity = 0.424), with a distinct increase in risk observed once UACR exceeds the 15 mg/g threshold.

## DISCUSSION

In this study, we identified a higher prevalence of the TLP in adolescents with type 1 diabetes compared with age and sex‐matched nondiabetic siblings. While the primary between‐group‐difference was observed in carotid IMT, the presence of TLP within the type 1 diabetes cohort was predominantly associated with structural remodeling in the lower extremity arteries, particularly the CFA. Age and urinary albumin excretion were independent predictors of TLP. Vascular changes became evident once UACR exceeded 15 mg/g, even within the conventionally defined normoalbuminuric range.

### TLP and peripheral vascular remodeling

TLP represents a distinct sonographic feature within the intima‐media complex and has been associated with increased IMT and atherosclerotic changes in large arteries, including the carotid and femoral arteries^11^. Proposed histologic correlates include medial fibrosis and elastocalcinosis, which are linked to aging, metabolic dysfunction, and diabetes‐related vascular injury^14^, ^15^, ^16^. In our study, TLP prevalence reached 64% among adolescents with type 1 diabetes, which is substantially higher than estimates reported in general populations of ~20%, suggesting that this phenotype may be under‐recognized and merits careful ultrasonographic assessment in high‐risk youth. A key finding was that, within the type 1 diabetes cohort, differences between TLP‐positive and TLP‐negative participants were concentrated in the femoral territory, whereas carotid IMT did not reveal meaningful differences by TLP status. Moreover, TLP was detected in lower extremity arteries and was not observed in carotid segments in our cohort. This distribution reflects an early stage of vascular remodeling and the distinct susceptibility of different arterial beds. While the carotid artery is an elastic artery designed to buffer cardiac pulsatility, the femoral and popliteal arteries are predominantly muscular arteries with a media layer dominated by vascular smooth muscle cells. Muscular arteries exhibit a more aggressive fibroproliferative response to metabolic stress, oxidative injury, and low‐grade inflammation, leading to earlier medial fibrosis and elastocalcinosis. Since TLP corresponds to the visualization of the internal elastic lamina, it captures these incipient structural changes in muscular arteries before generalized quantitative IMT thickening becomes detectable in elastic vessels. Furthermore, lower extremity arteries are exposed to higher hydrostatic pressure and distinct patterns of pulsatile load related to upright posture and daily ambulation. These biomechanical forces may amplify diabetes‐related vascular injury in the femoral and popliteal segments, accelerating early structural remodeling even when carotid IMT changes remain modest. Diabetes is associated with a propensity toward medial arterial changes, including early elastocalcinosis and alterations of the internal elastic lamina, which are captured sonographically as TLP. This phenotype therefore represents an earlier stage of peripheral vascular remodeling that is not fully reflected by carotid IMT at this young age. We acknowledge that vascular remodeling is dynamic and evolves with longer diabetes duration. It is plausible that with longer follow‐up, carotid qualitative changes such as TLP could emerge as the disease progresses. Our cross‐sectional observations likely reflect an early time point in the natural history of diabetes‐related vascular injury, where the muscular arteries of the lower extremities serve as the primary site for initial structural manifestations.

### Low‐grade albuminuria and vascular damage

Albuminuria reflects systemic endothelial dysfunction and has been linked to vascular inflammation and atherosclerosis^17^, ^18^, ^19^. Although albuminuria has traditionally been defined using a cutoff of 30 mg/g or higher, accumulating evidence suggests that low‐grade elevations below this threshold are clinically meaningful^3^, ^20^, ^21^, ^22^, ^23^, ^24^, ^25^. In type 2 diabetes, UACR values in the 10–30 mg/g range have been associated with increased cardiovascular and all‐cause mortality^24^, and recent studies suggest that a threshold of 15 mg/g or higher predicts kidney disease progression even among individuals classified as normoalbuminuric^25^.

Our findings extend this evidence to adolescents with type 1 diabetes by demonstrating that UACR 15 mg/g or higher was independently associated with TLP. Importantly, this association remained statistically significant after multivariable adjustment, whereas the conventional cutoff of UACR 30 mg/g or higher was not significant in the fully adjusted model. This pattern strengthens the interpretation that TLP may serve as an earlier vascular marker aligned with low‐grade renal microvascular injury, and it supports reconsideration of risk stratification strategies that rely solely on the traditional 30 mg/g threshold in youth with type 1 diabetes.

### Clinical implications

Regression of microalbuminuria is feasible in type 1 diabetes, and early intervention may reduce both renal and vascular complications^26^. Annual screening for UACR is recommended^2^. Our results further suggest that incorporating targeted vascular ultrasound assessment for TLP could refine cardiovascular risk stratification in adolescents with type 1 diabetes. Individuals with both elevated UACR and TLP may represent a subgroup warranting intensified preventive strategies to mitigate early vascular injury and improve long‐term outcomes.

### Limitation

Several limitations of this study should be considered. First, the cross‐sectional design precludes the establishment of a causal relationship between low‐grade albuminuria and the development of the TLP. Although we observed a robust association, longitudinal studies are required to determine whether incipient renal injury precedes or co‐evolves with peripheral vascular remodeling. Second, our study did not record the total daily insulin dose for participants. In children and adolescents with type 1 diabetes, insulin requirements are frequently titrated to account for pubertal growth, physical activity, and dietary variability. Therefore, a single cross‐sectional recording of insulin dosage may not provide a reliable representation of chronic insulin exposure. Third, although we adjusted for HbA1c, we lacked data from continuous glucose monitoring. These metrics might offer a more comprehensive reflection of metabolic control than a single HbA1c measurement. Finally, our study population consisted exclusively of Taiwanese adolescents. Given the potential ethnic variations in vascular architecture and diabetes complications, the generalizability of our findings to other ethnic groups remains to be validated.

## CONCLUSIONS

This study demonstrates that the TLP is prevalent in adolescents with type 1 diabetes and is associated with increased lower extremity IMT, suggesting early peripheral vascular remodeling. Age and UACR were independent predictors of TLP, and a UACR threshold of 15 mg/g showed a stronger association than the conventional 30 mg/g cutoff, indicating that subclinical vascular injury may begin at lower levels of albumin excretion. Incorporating TLP assessment into clinical screening may improve identification of high‐risk adolescents who could benefit from earlier, intensified intervention.

## AUTHOR CONTRIBUTIONS

Jung‐Chi Hsu contributed to data analysis, interpretation, and manuscript drafting. Fu‐Sung Lo recruited type 1 diabetes patients from the outpatient clinic. Jiann‐Shing Jeng performed carotid and lower‐limb vascular ultrasound examinations. Wen‐Yu Tsai recruited type 1 diabetes patients from the outpatient clinic. Sandy Huey‐Jen Hsu conducted laboratory examinations. Hsiang Yang performed dental health assessments. Jau‐Yih Tsauo was responsible for musculoskeletal and physical fitness examinations. Tien‐Jyun Chang provided critical comments and manuscript editing. Ta‐Chen Su designed the study, supervised participant recruitment and data collection, contributed to data interpretation, and critically revised the manuscript. All authors read and approved the final version of the manuscript and agreed to be accountable for all aspects of the work.

## FUNDING

This study was supported by the National Science and Technology Council (NSTC‐114‐2314‐B‐860‐002), Taiwan.

## DISCLOSURE

The authors declare no conflict of interest.

Approval of the research protocol: This study was conducted in accordance with the principles of the Declaration of Helsinki. The research protocol was reviewed and approved by the Institutional Review Board of National Taiwan University Hospital.

Informed Consent: Written informed consent was obtained from all participants or their legal guardians prior to enrollment.

Approval date of Registry and the Registration No. of the study/trial: N/A.

Animal Studies: N/A.

## Data Availability

The data that support the findings of this study are available on request from the corresponding author. The data are not publicly available due to privacy or ethical restrictions.
